# Rapid Reactivation of Deep Subsurface Microbes in the Presence of C-1 Compounds

**DOI:** 10.3390/microorganisms3010017

**Published:** 2015-02-05

**Authors:** Pauliina Rajala, Malin Bomberg, Riikka Kietäväinen, Ilmo Kukkonen, Lasse Ahonen, Mari Nyyssönen, Merja Itävaara

**Affiliations:** 1 Technical Research Centre of Finland (VTT), Kemistintie 3/Tietotie 2, FI-02044, Espoo, Finland; E-Mails: malin.bomberg@vtt.fi (M.B.); mari.nyyssonen@vtt.fi (M.N.); merja.itavaara@vtt.fi (M.I.); 2 Geological Survey of Finland (GTK), P.O. Box 96, FI-02151, Espoo, Finland; E-Mails: riikka.kietavainen@gtk.fi (R.K); lasse.ahonen@gtk.fi (L.A.); 3 University of Helsinki, P.O. Box 33, University of Helsinki, FI-00014, Helsinki, Finland; E-Mail: ilmo.kukkonen@helsinki.fi

**Keywords:** C-1 carbon, methanotrophy, sulphate reduction, nitrate reduction, terrestrial deep biosphere, microbial activity

## Abstract

Microorganisms in the deep biosphere are believed to conduct little metabolic activity due to low nutrient availability in these environments. However, destructive penetration to long-isolated bedrock environments during construction of underground waste repositories can lead to increased nutrient availability and potentially affect the long-term stability of the repository systems, Here, we studied how microorganisms present in fracture fluid from a depth of 500 m in Outokumpu, Finland, respond to simple carbon compounds (C-1 compounds) in the presence or absence of sulphate as an electron acceptor. C-1 compounds such as methane and methanol are important intermediates in the deep subsurface carbon cycle, and electron acceptors such as sulphate are critical components of oxidation processes. Fracture fluid samples were incubated *in vitro* with either methane or methanol in the presence or absence of sulphate as an electron acceptor. Metabolic response was measured by staining the microbial cells with fluorescent dyes that indicate metabolic activity and transcriptional response with RT-qPCR. Our results show that deep subsurface microbes exist in dormant states but rapidly reactivate their transcription and respiration systems in the presence of C-1 substrates, particularly methane. Microbial activity was further enhanced by the addition of sulphate as an electron acceptor. Sulphate- and nitrate-reducing microbes were particularly responsive to the addition of C-1 compounds and sulphate. These taxa are common in deep biosphere environments and may be affected by conditions disturbed by bedrock intrusion, as from drilling and excavation for long-term storage of hazardous waste.

## 1. Introduction

Deep environments in terrestrial bedrock have been considered potential sites for long-term storage of hazardous waste such as toxic metals and spent nuclear fuel because they are relatively stable, impermeable, nutrient limited, and show little biological activity [1]. However, highly diverse and numerous populations (10^3^–10^6^ cells mL^−1^) of microorganisms have been detected hundreds to thousands of metres below the surface in sedimentary, metamorphic and igneous rocks [2,3,4,5]. Although several studies have described the composition of these microbial communities [2,3,4,5], their ecology and activity remain unknown. Understanding the metabolic activity of these communities and their response to changing environmental conditions is of profound importance to the long-term safety assessments of hazardous waste disposal in deep subsurface sites. Studying these environments is challenging due to the technical difficulties involved in accurate sampling. New methods are needed to determine substrate specificities and activity rates of microbes and the biochemical processes they can catalyse or create if energy sources become available.

Construction in deep geological environments sites requires drilling into and excavation of bedrock environments. This can create new connections among otherwise isolated aquifers and affect native microbial processes due to the introduction of new carbon sources, e.g., C-1 compounds such as methanol and methane, and electron acceptors from sulphate-rich groundwaters. This may enable the processes that otherwise would be limited by scarcity of substrates and electron acceptors.

Methanol is used by a wide variety of microorganisms, whereas methane oxidation is a more uncommon feature [6]. Aerobic methanotrophic bacteria (MOB) oxidize methane to methanol by use of their methane monooxygenase enzyme (MMO) and, although these bacteria are mainly found in the presence of oxygen, they have been isolated from anaerobic environments in the deep biosphere [7]. While the role of MOB in anaerobic deep subsurface environments is not well understood, several strains of MOB have been isolated from Fennoscandian Shield sites [8].

A distinct lineage of nitrate reducing bacteria (NRB), *Methylomirabilis oxyfera*, can also perform a type of anaerobic oxidation of methane (AOM) via intra-cellular aerobic methane oxidation under anaerobic conditions [9]. Specific groups of archaeal anaerobic methanotrophs (ANME) may perform AOM via reversed methanogenesis [10]. Hydrogen released during AOM must be removed by reduction of electron acceptors such as sulphate (SO_4_^2−^) [10]. Sulphate-reducing bacteria (SRB) are known to function as syntrophic partners in AOM consortia [10]. In deep subsurface environments, SO_4_^2−^ is utilized as a “last resort” terminal electron acceptor in redox pathways, but its role in AOM is poorly understood. 

The deep subsurface microbial community of Outokumpu, Finland, has previously been investigated through the use of the deep Outokumpu drill hole, which has been drilled for scientific purposes [5,11]. The Outokumpu deep drill hole spans several isolated fracture zones. The fracture zone at a depth of 500 m contains saline groundwater with low sulphate concentration, and methane is the most abundant carbon source. Other terminal electron acceptors than sulphate have not been found. The bacterial community at this depth has been shown to comprise mostly α-proteobacteria, β-proteobacteria and Mollicutes, while the archaeal community is dominated by Methanobacteraceae [12]. Sulphate reducing, nitrate reducing and methane oxidizing bacteria have been detected in Outokumpu deep biosphere, but only at low abundances [12,13]. The low abundance of these specific functional bacterial groups may be due to unfavourable limiting environmental conditions, *i.e.*, low electron acceptor concentration. However, infiltration of water from, e.g., surface environments due to ground shifting or construction activities may change the equilibrium in formerly isolated deep subsurface aquifers.

In the present study, we focused on the bacterial community of the fracture zone at 500 m depth in Outokumpu. Because methane is so abundant (*i.e.*, ca. 75% of the dissolved gas) and is the principal carbon source available to microbes at this depth and sulphate is the main available electron acceptor [11] (Supplementary Text), we examined the preferences of fracture fluid microbes for methane or methanol as a carbon source and sulphate as an electron donor. The response of the microbial community to the C-1 compounds and/or sulphate was detected by molecular techniques that measure the metabolic activity within microbial cells. After exposure to substrates, the proportion of the microbial community responding to each carbon source or electron acceptor was determined using epifluorescence microscopy and a fluorescent dye that indicates redox activity in the cells. Transcription of specific genes during substrate induction trials was also evaluated with quantitative PCR (qPCR). Furthermore, the diversity of functionally relevant microbial groups was studied via denaturing gradient gel electrophoresis (DGGE) or with clone libraries, followed by sequencing of the genes detected.

## 2. Materials and Methods

### 2.1. Description of the Sampling Site

The Outokumpu deep borehole is situated in Outokumpu, eastern Finland (62°43′04″ N, 29°3′43″ E), in a Palaeoproterozoic sequence consisting of metasediments, ophiolite-derived altered ultramafic rocks and pegmatitic granite [14,15]. The Outokumpu mica gneiss- and granite-dominated rock association represents typical Fennoscandian bedrock. A 22 cm wide borehole was drilled in 2004–2005 to a total depth of 2516 m and spans several independent bedrock fracture zones [11,15].

### 2.2. Sample Collection

Sampling was performed in October 2010 and focused on the fracture zone at 500 m depth. This gas- and salt-rich fracture zone was isolated from the rest of the borehole with inflatable rubber packers (Lapela Oy, Rauma, Finland) as described in Ahonen *et al.* [14] and Purkamo *et al.* [12]. Packers were placed 24 m apart and enclosed a volume of 912 L of the borehole. The average yield of fluid from the 500 m fracture zone was 8.6 L h^−1^. After installation of the packer system, pumping continued for three weeks prior to sampling in order to ensure that samples represented authentic fracture fluid [12].

Fluid was pumped directly from the isolated fracture zone into an anaerobic glove box (GB-2202-S, MBRAUN, Garching, Germany) through a sterile polyamide tube. Anoxic conditions within the glove box were maintained with constant N_2_ (99.999%) flow and anaerobic generators (Anaerocult^®^ A, Merck, Darmstadt, Germany). In the anaerobic glove box, the microbial biomass from 1 L water samples was collected on polyethersulfone-filters of 0.2 μm pore size (PES) (Corning, Tewksbury, MA, USA) and exposed to a DNA and RNA isolation protocol. Three replicate samples for DNA and RNA were collected. In order to preserve the microbial mRNA, the sample processing time was kept short and filters were frozen on dry ice immediately after sample collection and maintained at −80 °C until nucleic acid isolation. In addition, fracture fluid for substrate injection experiments was collected in acid-washed, sterile, N_2_-flushed 100 mL glass serum bottles (Wheaton, NJ, USA) and 2 L Schott bottles (Duran group, Main, Germany), which were sealed with butyl rubber stoppers (Bellco Glass Inc., Vineland, NJ, USA) and aluminium open top crimp caps (Bellco Glass Inc., Vineland, NJ, USA) or plastic open top screw caps (Duran group, Main, Germany) to permit subsequent anaerobic sampling from the bottles. All water samples were stored at 6 °C and protected from light until analysed. The total cell number was determined within 48 h after sampling. Fracture fluid intended for substrate induction was stored at 6 °C for 3–4 weeks in order to ensure carbon deprivation.

### 2.3. Substrate Induction of Microbial Activity

The microbial response to different C-1 carbon substrates was examined by injecting CH_4_ (2.5 mL mL^−1^ fracture fluid) or CH_3_OH (10 μL mL^−1^ fracture fluid) to aliquots of anoxic sample water (Table S1). SO_4_^2−^ (in the form of Na_2_SO_4_ (532.8 μg mL^−1^ fracture fluid)) was also added to investigate the effects of an electron acceptor (Table S1). In order to minimize oxygenation of the samples due to injected fluids, all liquid supplements were flushed with N_2_ for 2 h prior to sterilisation. Sample fluid was divided into 12 subsamples (500 mL each) that were treated with four different substrate combinations (three parallel treatments/substrate combination) (Table S1). After the substrate injection, samples were incubated in a N_2_ atmosphere in 1 L sterile, acid-washed Schott bottles for 2 h at 18 °C in a shaker (45 rpm). Biomass from the incubated samples was collected on PES filters as described above for fracture fluid samples.

### 2.4. Staining of Microbial Cells

The total number of microbial cells was determined by 4,6-diamidino-2-phenylindole dye (DAPI) (Sigma, St. Louis, MO, USA) staining. Three parallel fracture fluid samples (10 mL) were stained with DAPI as described in Nyyssönen *et al.* [16]. Active microbial cells in the untreated fracture fluid and substrate-injected samples were stained with LIVE/DEAD^®^ *Bac*Light™ Bacterial Viability (L/D) as recommended by the manufacturer (Life Technologies, Grand Island, NY, USA), with an incubation time of 30 min. Fluid samples (two parallel samples of 10 mL) were anaerobically incubated in glass serum bottles with different substrate combinations (Table S1) for 2 h at 18 °C on a shaker (45 rpm) in a N_2_ atmosphere.

### 2.5. Microscopy and Cell Counting

Stained samples (DAPI, L/D) were concentrated on black 0.2 μm pore-size polycarbonate membrane filters (Isopore™ Membrane filters, 0.2 μm GTBP, Millipore, Danvers, MA, USA) with a Millipore 1225 Sampling Manifold (Millipore, Danvers, MA, USA) using low vacuum suction. The filters were examined under UV light and an epifluorescence microscope (Olympus BX60, Olympus Optical Ltd., Tokyo, Japan) at 100× magnification. Cells were counted in the field of view at 30 randomly chosen locations on the filter. The number of cells in the sample was calculated on the basis of magnification factor, filtered sample volume, and the active and total surface area of the filter as described by Nyyssönen *et al.* [17].

### 2.6. Nucleic Acid Extraction

DNA was extracted from the PES filters containing the microbial biomass of 500 or 1000 mL samples with the PowerWater DNA Isolation kit (MoBio Laboratories, Inc., Carlsbad, CA, USA) in accordance with the manufacturer’s protocol. RNA from activated or untreated samples was extracted from filter membranes with the PowerWater RNA Isolation kit (MoBio Laboratories, Inc., Carlsbad, CA, USA) in accordance with the manufacturer’s protocol. Nucleic acids were eluted with 50 or 100 μL of molecular grade water (500 and 1000 mL samples, respectively). Negative reagent controls for nucleic acid extractions were included in each extraction. Nucleic acids were stored at −80 °C until processing.

### 2.7. Reverse Transcription

DNA contamination of RNA extractions was checked by PCR amplification of a portion of 16S rRNA. PCR was performed in 50 μL reaction volumes containing 4 μL template, 1 × Dynazyme Reaction Buffer (Finnzymes, Vantaa, Finland), dNTP 62.5 μM each (final concentration) (Finnzymes, Vantaa, Finland), 0.2 μM (final concentration) of forward and reverse primers (Eurogentec, Seraing, Belgium) (Table 1), 1 U Dynazyme II polymerase (Finnzymes, Vantaa, Finland) and nuclease free H_2_O (Sigma, St. Louis, MO, USA). All PCR reactions were carried out in a Mastercycler gradient temperature cycler (Eppendorf, Hamburg, Germany) using the following conditions, 95 °C initial denaturation for 5 min followed by 40 cycles of 94 °C for 1 min, 55 °C for 1 min and 72 °C for 1 min, with a final extension step at 72 °C for 10 min.

The extracted RNA was converted to cDNA by reverse transcriptase-PCR (RT-PCR) using the Superscript III First Strand Synthesis SuperMix (Invitrogen, Carlsbad, CA, USA). RT-PCR was performed in two phases as instructed by the manufacturer. The cDNA was stored at −20 °C until further analysis.

### 2.8. Quantitative PCR

qPCR was used to determine the amount of *pmo*A, *dsr*B, *mcr*A, *mxa*F and *nar*G gene copies or transcripts in each sample. Three replicate samples of each treatment were each run in triplicate measurements. qPCR was performed in 10 μL reaction volumes using a LightCycler 480 qPCR machine and LightCycler 480 Software 1.5.0 (Roche Applied Science, Manheim, Germany). The reaction mixture contained 1 μL template, standard dilution or water, 1 × KAPA SYBR^®^ FAST Universal qPCR Master Mix (KAPA Biosystems, Wilmington, MA, USA), 2.5 μM of each forward and reverse primer (Table 1) and nuclease free water. As template, DNA and cDNA from fracture water as well as cDNA from each of the induced samples were used. Nuclease free water was used as a negative control in PCR. A 10-fold plasmid dilution series containing 10^1^–10^7^ copies of the relevant gene insert was used as standard. The PCR program consisted of an initial 15 min incubation at 95 °C, followed by 45 cycles of denaturation at 95 °C for 10 s, annealing at 55 °C for 35 s and extension of 72 °C for 30 s, and with final extension at 72 °C for 3 min. Sample fluorescence was measured at the end of each elongation phase. Subsequently, a melting curve was recorded to test the specificity of the qPCR, with a program consisting of a 10 s denaturation at 95 °C, 1 min of annealing at 65 °C, and a melting and continuous measuring step rising gradually (0.11 °C s^−1^) to 95 °C. For qPCR the efficiency 95%–103% was accepted.

### 2.9. PCR

*pmo*A, *mxa*F, *dsr*B and *nar*G gene fragments were amplified from the three parallel samples of DNA and cDNA obtained from untreated fracture fluid as well as from the treated samples. PCR was performed in 50 μL reaction volumes containing 4 μL template, 1 × Dynazyme Reaction Buffer (Finnzymes, Vantaa, Finland), 62.5 μM dNTP (final concentration) (Finnzymes, Vantaa, Finland), 0.2 μM (final concentration) of each forward and reverse primer (Eurogentec, Belgium) (Table 1), 1 U Dynazyme II polymerase (Finnzymes, Vantaa, Finland) and nuclease free water (Sigma, St. Louis, MO, USA). All PCR reactions were carried out on a Mastercycler gradient temperature cycler (Eppendorf, Hamburg, Germany) using the following conditions, 95 °C initial denaturation for 5 min followed by 40 cycles of 94 °C for 1 min, 55 °C for 1 min and 72 °C for 1 min, with final extension step at 72 °C for 10 min, with the exception of an annealing temperature of 58 °C being used for *nar*G. PCR products were visualized with agarose gel electrophoresis on a 1% agarose gel (LE-agarose, Lonza, Basel, Switzerland) stained with 1 × SyberSafe (Invitrogen, Carlsbad, CA, USA) in 1 × SB-buffer [18] at 300 V for 15 min.

### 2.10. DGGE Analysis of SRB

SRB species diversity was investigated by PCR-DGGE analysis of the *dsr*B gene fragment. *dsr*B amplicons were resolved by DGGE on 8% acrylamide with a 40%–70% denaturing gradient electrophoresed at constant voltage of 85 V and a temperature at 60 °C for 20 hours in 0.5 × TAE running buffer. Gels were visualized and DGGE bands were excised and prepared for sequencing according to Nyyssönen *et al.* [17]. DNA fragments were sequenced at Macrogen Inc. (Seoul, Korea) with the DSR4R primer (Table 1).

### 2.11. Methanotroph and NRB Diversity According to Clone Libraries

The DNA fragments from *pmo*A- and *nar*G-targeted PCR were excised from the agarose gel and purified with the Qiaquick Gel Extraction kit (Qiagen, Hilden, Germany). PCR amplicons were ligated into the pCR^®^ 2.1-TOPO vector using the TOPO TA Cloning kit (Invitrogen, Carlsbad, CA, USA) according to the manufacturer’s instructions. Chemically competent One Shot TOP10F Competent Cells (Invitrogen, Carlsbad, CA, USA) were transformed with 2 μL of ligation mixture according to the manufacturer’s instructions. Transformed clones were grown overnight at 37 °C on Luria-Bertani (LB) agar plates containing kanamycin (50 μg mL^−1^) as a selective agent. Successful insertion of the vector was verified by testing 96 clones of each treatment via colony PCR. A small amount of each colony was added as template to each PCR and simultaneously transferred to a fresh LB-kanamycin plate. Colony PCR was performed with M13f and M13r primers (Invitrogen, Carlsbad, CA, USA) in 50 μL reactions containing 1 × Green GoTaq Flexi buffer (Promega, Madison, WI, USA), 100 μM dNTP (Finnzymes, Vantaa, Finland), 1.5 mM MgCl_2_, 0.2 μM of each M13 forward and reverse primer, 1 U GoTaq polymerase (Promega, Madison, WI, USA) and nuclease free water (Sigma, St. Louis, MO, USA). PCR was carried out in a Mastercycler gradient temperature cycler (Eppendorf, Hamburg, Germany) and the following conditions: 95 °C denaturation for 5 min followed by 40 cycles of 94 °C for 1 min, 50 °C for 1 min and 72 °C for 1 min, with a final extension step at 72 °C for 7 min. PCR products were visualized on a 1% agarose gel (as described above) and later sequenced at Macrogen Inc. (Seoul, Korea) with the T7 primer.

### 2.12. Sequence Analysis, Phylogeny and Statistical Analyses

Sequences obtained from the clone libraries and DGGE were imported into the Geneious Pro Software package (version 5.3.6, Biomatters Inc., Auckland, New Zealand) [19] where they were manually checked, assembled and edited prior to phylogenetic analysis. Possible vector contamination was cleaned with the UniVec-tool in Geneious Pro. Sequences were compared to those in public databases via a BLAST search [20]. The closest matching sequences, relevant reference sequences and sequences of type species of each gene type were included in the phylogenetic analyses. Sequences were converted to amino acid residues and aligned using ClustalW [21] with default parameters in Geneious Pro. All alignments were checked and manually edited. Maximum likelihood analysis was performed on the amino acid sequence alignments using PhyML [22] and the Whelan and Goldman (WAG) substitution model [23]. Nonparametric bootstrap support was calculated for the recovered nodes with 1000 pseudoreplicates. Due to the high number of similar *nar*G sequences obtained from clone libraries, terminals sharing over 99% of the amino acid sequence were collapsed into single OTUs and a single sequence was chosen to represent the OTU in subsequent analyses.

Cochran’s Q test, one-way analysis of variance and subsequent multiple comparisons analysis using Tuckey’s variance was performed for qPCR results using the SPSS software (v15.0, SPSS Inc., Chicago, IL, USA).

### 2.13. Accession Numbers 

Sequences were submitted to European Nucleotide Archive with accession numbers *ERS653256- ERS653340.*

## 3. Results

### 3.1. Microbial Response to Substrate

**Figure 1:**
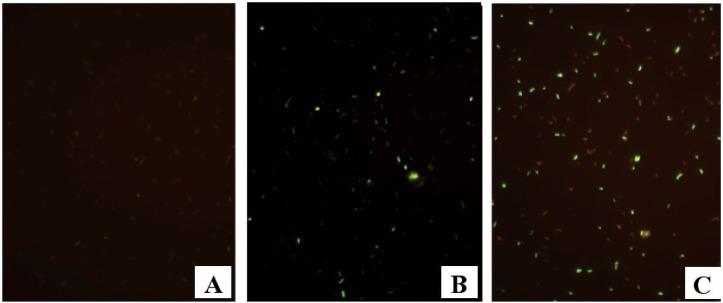
Epifluorescence micrographs of L/D stained samples, where green reflects metabolic activity and red reflects inactivity. (**A**) Untreated fracture water (**B**) CH_4_ induced (**C**) CH_4_ + SO_4_^2−^ induced.

**Figure 2:**
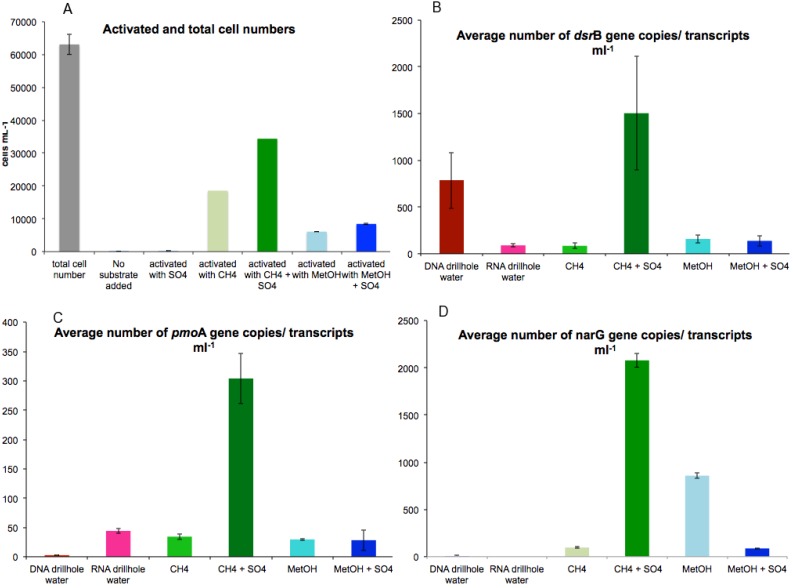
(**A**) Mean concentrations of microbial cells detected with DAPI and substrate-induced cells detected by Live/Dead staining, and (**B**–**D**) gene copies and transcripts mL^−1^ fracture fluid. Error bars represent standard error of mean (*n* = 3).

The response of microbial communities to the addition of methane (CH_4_) or methanol (CH_3_OH) in the presence or absence of the electron acceptor (SO_4_^2−^) is shown in Figure 1A–C. Cell metabolic activity was induced by injecting CH_4_ or CH_3_OH with and without SO_4_^2−^ to the fracture fluid sample (Table S1). The total cell number in the untreated fracture fluid was 6.3 × 10^5^ cell mL^−1^, as determined by DAPI staining and subsequent microscopy. Of these cells 0.3% appeared active when examined microscopically with the Live/Dead stain (Figure 2A). CH_4_ + SO_4_ had the greatest impact, causing 60% of microbial cells (compared to DAPI stained untreated sample) to increase their metabolic activity (Figure 2A). Without SO_4_^2−^, CH_4_ activated 30% and CH_3_OH 10% of microbial cells (Figure 2A). CH_3_OH + SO_4_^2−^ activated 13% of microbial cells (Figure 2A). SO_4_^2−^ without a C-1 substrate did not affect microbial activity (Figure 2A).

### 3.2. Activation of Functional Genes by C-1 Compounds

Quantitative PCR (qPCR) was used to determine the copy number and amount of transcripts for five functional genes in the fracture fluid samples. SRB were studied using the primers targeting the β-subunit of dissimilatory sulfite reductase (*dsr*B) (Table 1). The number of *dsr*B genes in the untreated fracture fluid was significantly higher, 7.8 × 10^2^ gene copies mL^−1^ (*p* < 0.01), than the number of transcripts, 9 × 10^1^ mL^−1^, indicating that the SRB in the untreated fracture water were quite inactive (Figure 2B). Addition of CH_4_ + SO_4_^2−^ significantly increased the number of *dsr*B transcripts to a 100-fold (1.5 × 10^3^ transcripts mL^−1^ fracture fluid, *p* < 0.05) (Figure 2B), whereas the addition of CH_3_OH and CH_3_OH + SO_4_^2−^ induced a 10-fold increase (1.5 × 10^2^ transcripts mL^−1^) compared to untreated fracture fluid (Figure 2B). Addition of CH_4_ alone did not affect the transcription of *dsr*B in comparison to untreated fracture fluid (Figure 2B). In agreement with the activity assays, addition of SO_4_ alone did not affect to the transcription activity of the *dsr*B genes.

**Table 1:** Primers used for screening *mxa*F, *pmo*A, *dsr*B and *mcr*A and genes.

Gene (bp)	Primer	Reference
mxaF 557 bp	F100/R1561	[24]
pmoA 330 bp	pmof1/pmor	[25]
dsrB 370 bp	DSRp2060F/DSR4R	[26,27]
mcrA 330 bp	ME1/ME3	[28]
narG 110 bp	1960m2f/2050m2r	[29]

The response of NRB was studied using primers targeting the α-subunit of the nitrate reductase (*nar*G) (Table 1). The number of *nar*G genes and transcripts in the fracture fluid were beneath the assay detection limit. However, the addition of CH_4_ + SO_4_^2−^ stimulated the transcription of *nar*G showing a significant increase in the number of *nar*G transcripts of up to 2.1 × 10^3^ transcripts mL^−1^ (*p* < 0.01) (Figure 2C). Addition of CH_3_OH also significantly increased the transcription of *nar*G to 8.6 × 10^2^ transcripts mL^−1^ (*p* < 0.05) compared to the untreated fracture fluid. The smallest effect was detected with the addition of CH_4_ or CH_3_OH + SO_4_^2−^, 9 × 10^1^ *nar*G transcripts mL^−1^ (Figure 2C).

The effect of C-1 compounds on MOB was studied by targeting the α-subunit of the particulate methane monooxygenase (*pmo*A) (Table 1). The number of *pmo*A genes and transcripts in the fracture fluid prior to substrate induction was below the detection limit of the qPCR assay (Figure 2D). After induction, the number of *pmo*A transcripts increased significantly to 3 × 10^2^ transcripts mL^−1^ in samples treated with CH_4_ + SO_4_^2−^ (Figure 2D) (*p* < 0.05). Other treatments showed 10-fold fewer *pmo*A transcripts (Figure 2D).

The amount of methanogens and methylotrophs, detected via qPCR of the α-subunit of methyl coenzyme M reductase (*mcr*A) or methanol dehydrogenase (*mxa*F), respectively, were below the detection limit (<10 copies mL^−1^) in all samples and treatments, or were not detectable by the primers used in this study.

### 3.3. Sulphate Reducers

**Figure 3:**
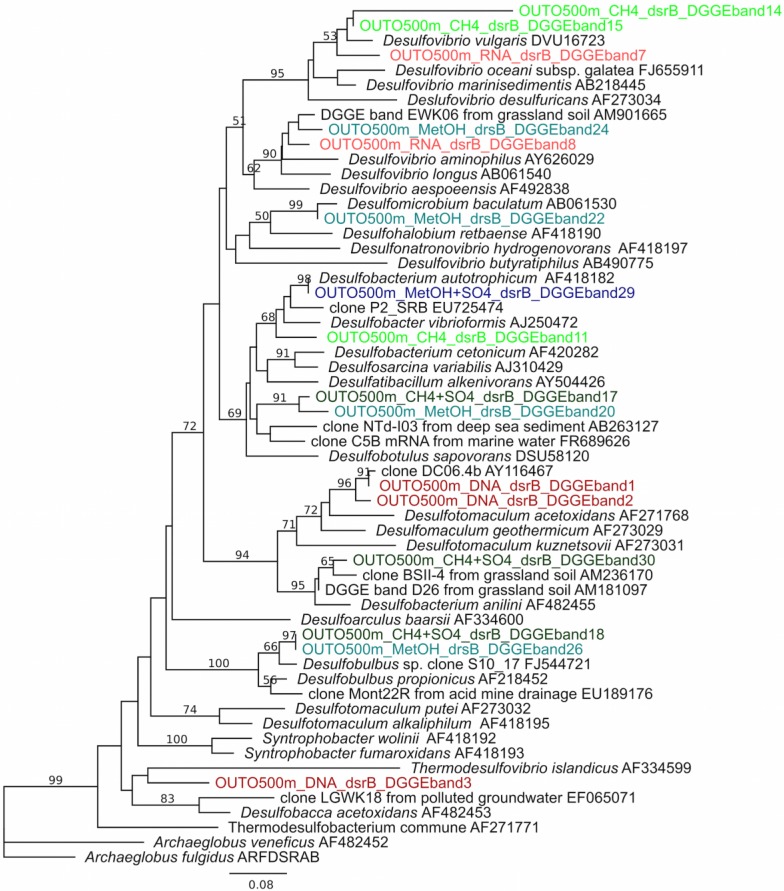
Phylogenetic tree of SRB based on *dsr*B amino acid sequences obtained by PCR-DGGE and in relation to cultured SRB and the closest uncultured relatives. The *dsr*B sequences from DNA and RNA (directly obtained from untreated fracture fluid) and substrate induced samples are represented in different colours, DNA—red, RNA—light red, CH_3_OH—light blue, CH_3_OH + SO_4_^2−^—blue, CH_4_—light green and CH_4_ + SO_4_^2−^—green. The band number indicates the DGGE band number in Fig. S1. Nonparametric bootstrap values are shown at nodes found in >50% of 1000 pseudoreplicates. The scale bar indicates 0.08 amino acid substitutions. The tree is rooted by *Archaeglobus veneficus.*

Diversity of SRB in untreated and treated fracture fluids and the transcription of *dsr*B genes were investigated with *dsr*B-based DGGE (Figure S1). The DGGE analysis revealed 13 different *dsr*B OTUs (Figure S1). Firmicutes appeared to be the most abundant SRB in untreated fracture fluid, but were only detected from DNA. *dsr*B transcripts in the untreated fracture water belonged to δ-Proteobacteria, e.g., *Desulfovibrio* sp. (Figure 3) and no transcripts resembling Firmicutes were detected. Transcription of *dsr*B of δ-Proteobacteria was clearly induced by C-1 substrates and resulted in a more diverse set of transcripts compared the to untreated fracture fluid, *i.e.*, belonging to genera *Desulfobulbus*, *Desulfobacterium* and *Desulfovibrio* (Figure 3). A maximum of five OTUs were obtained after CH_4_ and CH_3_OH induction (Figure S1). A total of four OTUs were obtained after CH_4_ + SO_4_^2−^ induction and RNA extracted from untreated fracture fluid.

### 3.4. Nitrate Reducers

The diversity and identity of NRB species activated by CH_4_ + SO_4_^2−^ and CH_3_OH was studied with clone libraries. *nar*G transcripts detected after CH_4_ + SO_4_^2−^ induction clustered in five OTUs and transcripts detected after CH_3_OH induction formed one OTU (99.7%–100% sequence similarity within an OTU, Table S2). In the phylogenetic analysis, the OTUs fell into one tight cluster (Figure S2). All OTUs were mainly affiliated with *nar*G sequences of γ-proteobacterial species (group I) (Figure S2).

### 3.5. Methanotrophs

Clone libraries of *pmo*A genes and transcripts were obtained from DNA and RNA isolated directly from the untreated fracture fluid and from all samples receiving substrate. All sequenced clones resembled the γ-proteobacterial type X methanotrophs (Figure S3).

### 3.6. Methanogens and Methylotrophs

No *mcr*A or *mxa*F genes or transcripts of methanogens and methylotrophs, respectively, were detected in the original fracture water or in any of the treatments.

## 4. Discussion

Construction of underground waste repositories requires destructive penetration to long-isolated bedrock environments. Such activity will likely introduce carbon sources and electron acceptors that are novel to the native microbial flora and its ecology. Furthermore, destructive intrusion of bedrock systems force previously isolated aquifers to mix, leading to new interactions among microbial communities and their energy sources.

In the present study, we developed a method based on substrate induction to study the response of microbes from deep terrestrial subsurface to changing conditions in their environment. In accordance with the findings of D’Hont *et al.* [30] and Parkes *et al.* [31] in deep-sea sediments, our metabolic and transcriptional activity analyses demonstrate a low metabolic activity of microorganisms at a fracture zone 500 m below the surface of the Fennoscandian Shield. However, a two-hour exposure to C-1 substrates is sufficient to trigger an increase in metabolic activity of the microbial community. Morono *et al.* [16] showed how microbes in deep-sea sediments that have been starved for hundreds of thousands of years and appear dead or inactive can readily incorporate carbon and nitrogen compounds into their biomass should the opportunity arise. In addition, our findings show that the deep subsurface microorganisms have the capability to respond to changing conditions in their habitat even more rapidly, within hours.

Our results show that CH_4_ addition together with SO_4_^2−^ had the strongest influence on the activity of the microbial community. This treatment induced the metabolic activity of over 60% of the microbial community in the fracture water in comparison to the 0.3% active cells in the untreated fracture water. This treatment also showed a simultaneous activation of the transcription of *pmo*A, *nar*G and *dsr*B genes of known aerobic methanotrophs (MOB), nitrate reducers (NRB) and sulphate reducers (SRB), respectively. 

Genes previously identified from aerobic CH_4_ oxidizing bacteria have been identified by metagenomic analyses of samples from the Outokumpu deep biosphere at 600 m depth [32]. Methane oxidizing bacteria identified by their *pmo*A genes or transcripts were also detected in all samples and treatments in this study. All identified *pmo*A genes and transcripts associated with the γ-proteobacterial group X methanotrophs. This is in agreement with previous studies at this site where *pmo*A genes from 600 m depth mostly resembled *Methylomonas methanica*, also a γ-proteobacterial methanotroph [13]. These methanotrophs were only found at specific depths of the borehole length, at 600, 900 and 1500 m depths, where either the inflow of fracture water or a major change in salinity appeared [13,32], *i.e.*, at the most dynamic parts of the borehole. The fact that γ-proteobacterial methanotrophs were abundant at these depths and that the otherwise scarce γ-proteobacteria activate so highly upon change in the environment indicates that this group of bacteria have the capacity to rapidly respond to environmental change. The mechanisms behind the activation of these generally aerobic CH_4_ oxidizing bacteria under these anaerobic conditions, especially in response to CH_4_ + SO_4_^2−^, remain elusive.

According to 16S rRNA data [16] there are no other known methane utilizers, such as *Verrucomicrobia* [33] or NC10 bacteria [9] at this depth in untreated borehole fluid. However, nitrite-dependant anaerobic methane oxidation cannot be completely excluded. No *mcr*A gene transcripts of ANME archaea were detected in any of the treatments, thus implying that γ-proteobacterial MOB, instead of archaeal ANME process, is a key microbial group oxidizing methane in Outokumpu groundwater.

NRB identified by their *nar*G genes could not be detected by qPCR in the fracture water at 500 m depth, indicating that NRB are few and inactive in this habitat. This is in agreement with Purkamo *et al.* [13], who found only a marginal amount of *nar*G gene copies in the Outokumpu deep borehole water column. In addition, nitrate was not detected from the borehole water or fracture water or else it was present at concentrations below the limit of detection. However, the salinity of the fracture water increases the detection limit to 100 mg nitrate L^−1^. Thus, we cannot with certainty exclude the presence of nitrate. Nevertheless, our results show that the NRB present in Outokumpu can activate rapidly when suitable conditions arise. The role of NRB in this system is not known and their reaction to the addition of CH_4_ + SO_4_^2−^ especially is not clear. Are the NRB able to use the nitrate reductase for sulphate reduction? The majority of the detected NRB were similar to *Hahella chejuensis*, a marine anaerobic γ-proteobacterium [34]. Thus, despite being only a marginal group in the total microbial community in Outokumpu, these bacteria were also rapidly activated due to changes in their living environment. If anaerobic methane oxidation mediated by sulphate reduction was activated in our microcosms it is possible that the resulting sulphide activated the NRB, although only trace nitrate would be present [35]. This “co-activation” may play an important role in the biological potential of this habitat.

The SRB reacted to the substrate additions in the same way as the MOB and NRB, showing a peak in the transcription of the *dsr*B gene in response to the CH_4_ + SO_4_^2−^ treatment. SRB, especially δ-proteobacteria belonging to Desulfobacteraceae and Desulfobulbaceae, have been identified as bacterial partners for AOM in marine sediments [36] where they facilitate the anaerobic oxidation of CH_4_ by removing excess H^+^ [10]. Based on DGGE followed by sequencing, these SRB were the main groups responding to CH_4_ + SO_4_^2−^ in our study as well. Thus, our results agree with the suggested involvement of SRB in methane oxidation [10] and demonstrate that an entirely different and distinct subpopulation of δ-proteobacterial SRB is activated when suitable C-1 substrates become available. These δ-proteobacterial SRB are also commonly detected in Olkiluoto (South West Finland) deep bedrock fracture waters experiencing mixing of groundwater layers [17,37], where they appear to be part of the metabolic potential of the microbial reserve that is present at low concentrations in this habitat. Many microbial groups are being considered as biological indicators of the state of an environment, or a specific environmental process [38,39,40], and the microbial groups and processes described above could be potential indicators for the stability or change in deep repository environments.

The methanol dehydrogenase of methylotrophs was not found in this study, despite the activating effect of methanol on the microbial community. This may be due to primer specificity issues. The primers used in this study target the methanol dehydrogenase of type II methanotrophs [41]. Based on the *nar*G and *pmo*A genes found in this study, the methylotrophs in Outokumpu may rather be γ-proteobacterial and thus the *mxa*F primers used here may not be suitable for identifying them [24].

## 5. Conclusions

Microbial communities in terrestrial deep subsurface environments are relatively inactive due to limited availability of carbon substrates and suitable electron acceptors. Despite their apparent inactivity, we here show the rapid response of MOB, NRB and SRB to simple carbon compounds, such as methane and methanol, and the electron acceptor SO_4_^2−^.

A metabolic and transcriptional response of NRB to simultaneous availability of methane and SO_4_^2−^ suggests that they may be a key group bringing together the thus far little known processes surrounding CH_4_ utilization in deep subsurface environments. Since most of the typical microbial groups involved in AOM processes were not found in this study, NRB may play a much more important role in these communities than previously thought. NRB and SRB were clearly activated when simple one-carbon compounds were introduced and they may either utilize these substrates themselves or take part in syntrophic processes and thus benefit from these compounds secondarily.

Rapid revival of microorganisms in the deep biosphere in response to novel conditions is an important consideration for the safety and stability of underground waste repositories. This feature of microbial ecology should be taken into account for risk assessment associated with the long-term storage of hazardous waste in deep subsurface environments.

## Supplementary Files

Supplementary File 1
