# Supplementary material for: Rapid Reactivation of Deep Subsurface Microbes in the Presence of C-1 Compounds
Source: Microorganisms. 2015 Feb 5;3(1):17–33. doi: 10.3390/microorganisms3010017 (PMC5023232; doi:10.3390/microorganisms3010017)
Supplement: Supplementary File 1 [file microorganisms-03-00017-s001.docx]

**Supplementary Material**

Groundwater Chemistry

Electrical conductivity, pH, Eh, and concentration of dissolved oxygen (O_2_) were continuously monitored in the field during pumping with WTW sensors attached to a flow-through cell. In addition, fracture fluid samples were collected at least once a week and analysed for their chemical composition at Labtium Oy (Espoo, Finland). Fracture fluid samples for cation analysis were filtered in the field (Whatman FP, 0.45 μm, Whatman GmbH, Dassel, Germany) and acidified with 65% ultrapure HNO_3_
(5 μL mL^−1^) and measured by ICP-MS (Perking Elmer, Waltham, MA, USA and Agilent Technologies, Santa Clara, CA, USA) or ICP-OES (Thermo Jarrell Ash Corp., Franklin, MA, USA). Anions were determined from untreated samples by ion chromatography. End-point titration to pH 4.5 was used to determine alkalinity and the speciation of alkalinity was calculated using PHREEQC software [1]. Gas samples were collected from the released gas phase either by injecting or conveying the gas under sample water into Ar-flushed glass bottles, and analysed with a gas chromatograph at Ramboll Analytics (Vantaa, Finland).

Chemical composition of the fracture water stayed relatively constant during the whole pumping experiment. During the sampling in October 2010, the temperature of the water at 500 m depth was 12 °C, and the pH was 9.3. The electrical conductivity (EC) of the fracture water was 19.3 mS cm^−1^, corresponding to the salinity (TDS) of 12 g L^−1^, and mainly composed of calcium, sodium, and chloride. Sulphate was 0.7 mg L^−1^. Negative Eh values, down to −152 mV, confirmed strictly anaerobic conditions in the fracture fluid. Concentration of dissolved oxygen was below 0.1 mg L^−1^. 75% of the gas phase consisted of methane and 21% of nitrogen. Other gases detected were helium (2%), ethane (0.9%), argon (0.2%), propane (0.027%), and hydrogen (0.007%). Alkalinity of the fracture water was only
0.20 mmol L^−1^, indicative of low amount of dissolved inorganic carbon (DIC). The main constituent of DIC at the prevailing pH is CaCO_3_ followed by HCO_3_^−^.

**Table S1.** Substrates/substrate combinations added to samples prior to LIVE/DEAD, staining or activation of transcription.

| **Substrate** | **Microscopy Analyses** | **Activation of Transcription** |
| --- | --- | --- |
| no substrate control | no substrate added |  |
| SO_4_^2−^ | 5.55 μM | na |
| CH_4_ (99.995%) | 66.0 mM | 5.2 mM |
| CH_4_ (99.995%) + SO_4_^2−^ | 66.0 mM/5.55 μM | 5.2 / 5.55 μM |
| CH_3_OH | 246.6 μM | 246.6 μM |
| CH_3_OH + SO_4_^2−^ | 246.6 μM/5.55 μM | 246.6 μM/5.55 μM |

na, not analyzed.

**Table S2.** OTUs from *nar*G sequences.

| **OTU** | **Substrate** | **Number of Clones** | **Similarity of Sequences within OTU (%)** |
| --- | --- | --- | --- |
| 1 | CH_4_ + SO_4_^2−^ | 2 | 100 |
| 2 | CH_4_ + SO_4_^2−^ | 1 | 100 |
| 3 | CH_4_ + SO_4_^2−^ | 1 | 100 |
| 4 | CH_4_ + SO_4_^2−^ | 1 | 100 |
| 5 | CH_4_ + SO_4_^2−^ | 1 | 100 |
| 6 | CH_4_ + SO_4_^2−^ | 1 | 100 |
| 7 | CH_4_ + SO_4_^2−^ | 2 | 100 |
| 8 | CH_4_ + SO_4_^2−^ | 1 | 100 |
| 9 | CH_4_ + SO_4_^2−^ | 25 | 100 |
| 10 | CH_4_ + SO_4_^2−^ | 1 | 100 |
| 11 | CH_4_ + SO_4_^2−^ | 3 | 100 |
| 12 | CH_4_ + SO_4_^2−^ | 1 | 100 |
| 13 | CH_3_OH | 1 | 100 |
| 14 | CH_3_OH | 2 | 100 |
| 15 | CH_3_OH | 1 | 100 |
| 16 | CH_3_OH | 2 | 100 |
| 17 | CH_3_OH | 1 | 100 |
| 18 | CH_3_OH | 3 | 100 |
| 19 | CH_3_OH | 15 | 100 |
| 20 | CH_3_OH | 3 | 100 |
| 21 | CH_3_OH | 1 | 100 |
| 22 | CH_3_OH | 1 | 100 |
| 23 | CH_3_OH | 2 | 100 |
| 24 | CH_3_OH | 1 | 100 |


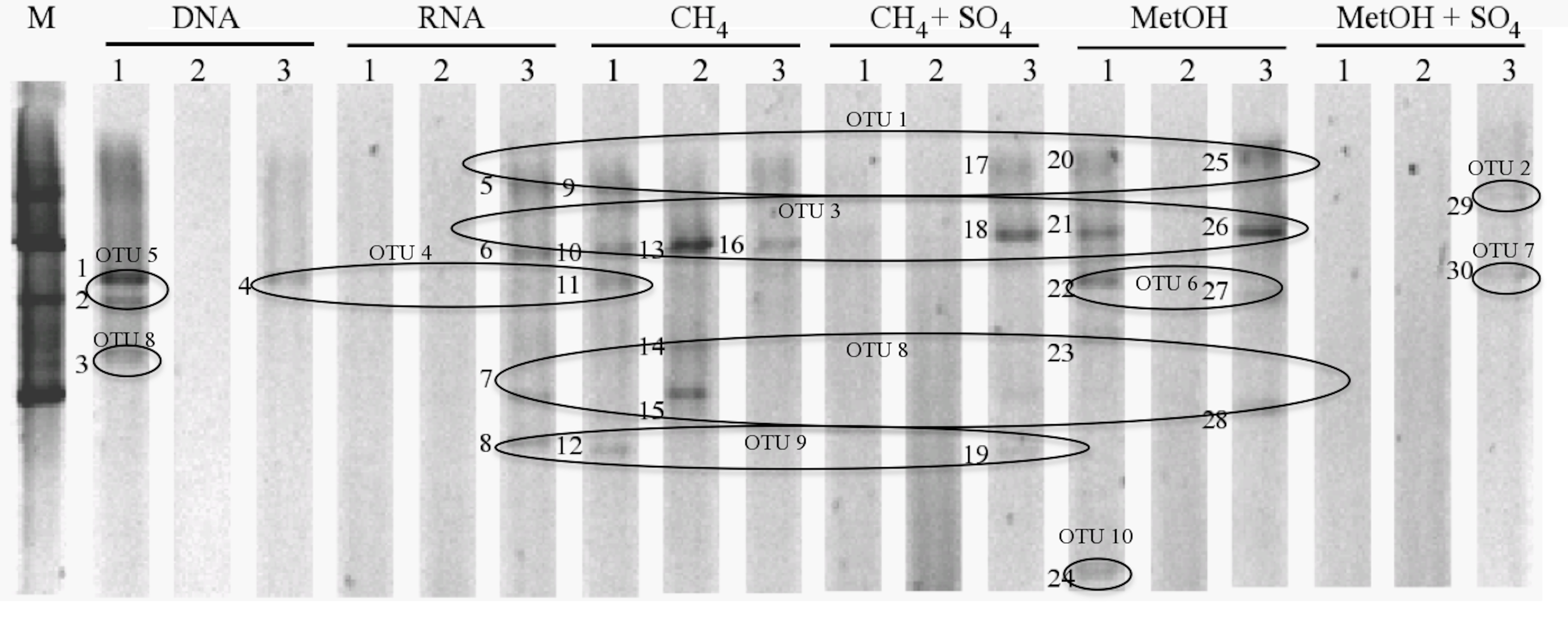


**Figure S1.** Detection of SRB diversity based on *dsr*B DGGE, where M is *dsr*B DGGE marker. The sample lanes from the left represent DNA and RNA from the untreated fracture water, CH_4_, CH_4_ + SO_4_, CH_3_OH and CH_3_OH + SO4 refer to the RNA extracted from fracture water treated with these substrates. Three replicate samples (1, 2, 3) were ran for each sample type and treatment. The numbered DGGE bands were sequenced.

**
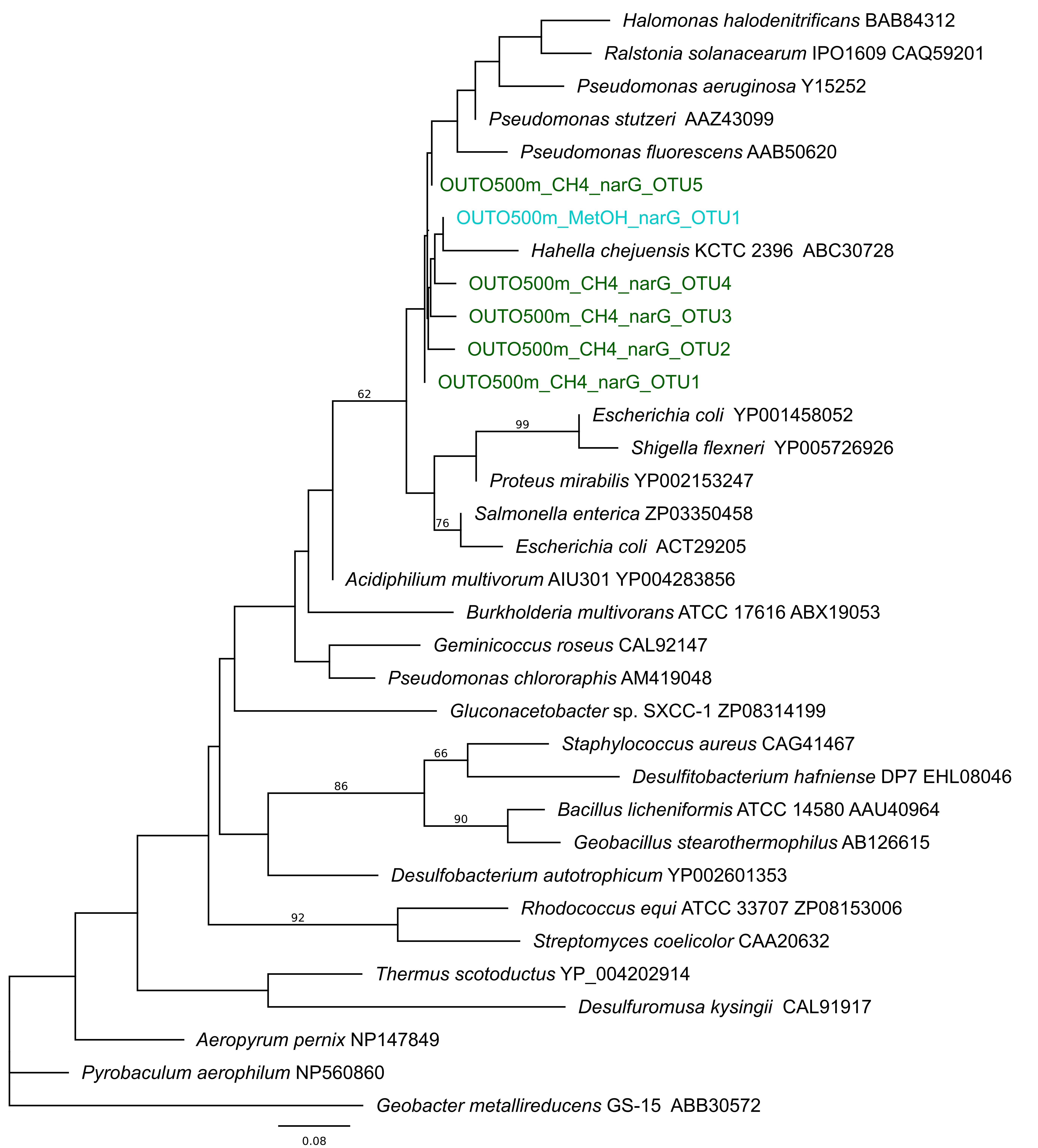
**

**Figure S2.** Phylogenetic tree of NRB based on the *nar*G sequences (translated to amino acid) obtained by *nar*G gene-based clone library, in relation to cultured NRB and the closest uncultured relatives. The *nar*G sequences from substrate induced samples are presented in different colors, Methanol (CH_3_OH)—light blue, and CH_4_ + SO_4_^2−^—green. Nonparametric bootstrap values for nodes found in >50% of 1000 pseudoreplicates are shown. Sequences from substrate activated samples are presented in different colors, CH_3_OH—light blue and CH_4_ + SO_4_^2−^—green. The scale bar indicates 0.08 amino acid substitutions. The tree is rooted with *Pyrobaculum aerophilum*.


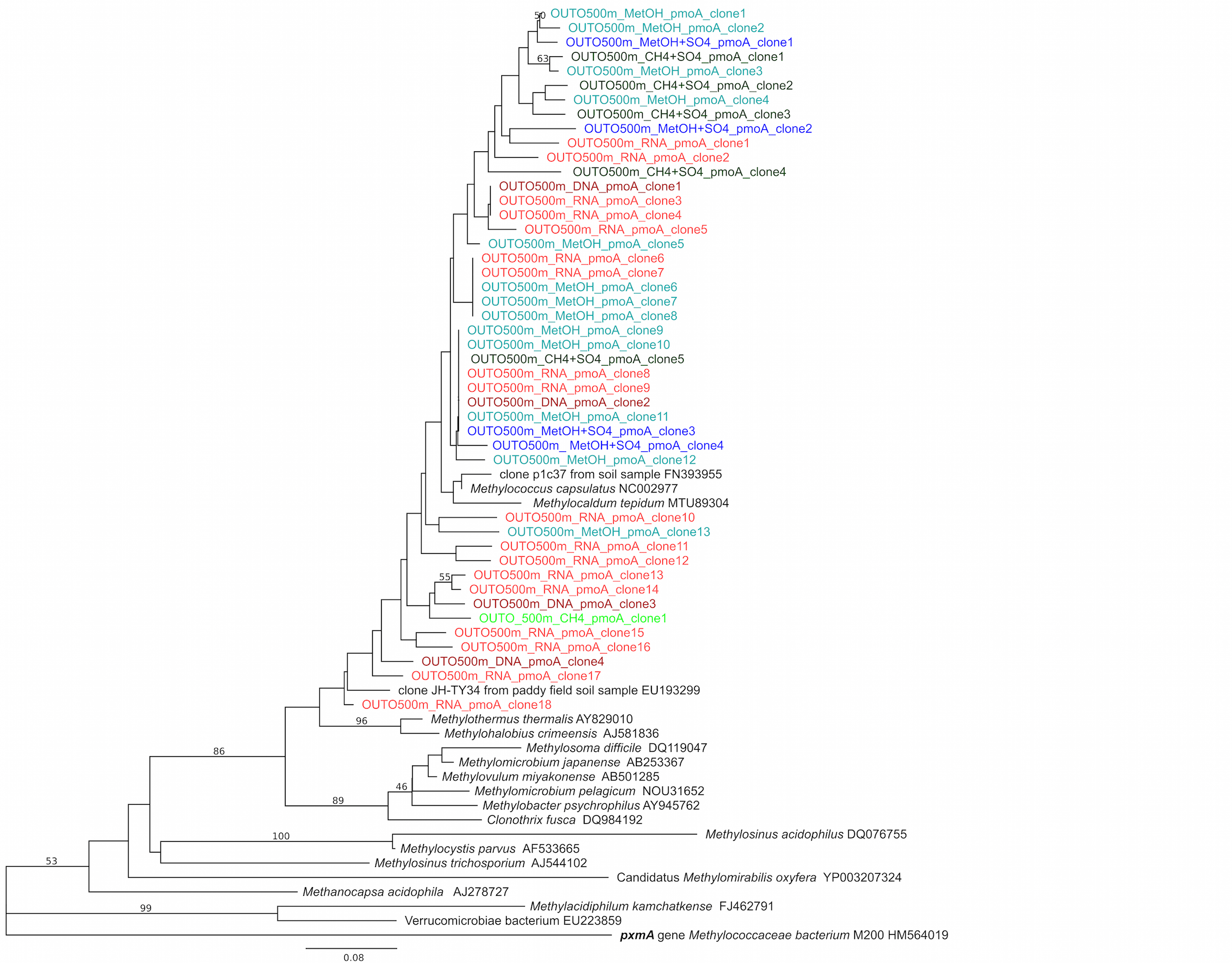


**Figure S3.** Phylogenetic tree of methanotrophs, based on the *pmoA* sequences (translated to amino acid) obtained by *pmo*A gene-based clone library in relation to cultured methanotrophs and the closest uncultured relatives. Sequences are *pmoA* unless otherwise labeled. The *pmo*A sequences from DNA and RNA (directly obtained from untreated fracture fluid) and substrate induced samples are presented in different colors, DNA—red, RNA—light red, Methanol (CH_3_OH)—light blue, CH_3_OH + SO_4_^2−^—blue, CH_4_—light green and CH_4_ + SO_4_^2−^—green. Nonparametric bootstrap values for nodes found in >50% of
1000 pseudoreplicates are shown. The scale bar indicates 0.08 amino acid substitutions. The tree is rooted by *pxm*A gene of *Methylococcaceae* bacterium.

References

1. USGS (2014) PHREEQC. Computer Codes, United States Geological Survey. Available online: http//wwwbrr.cr.usgs.gov/projects/GWC_coupled/phreeqc/ (accessed on 3 October 2014).

© 2015 by the authors; licensee MDPI, Basel, Switzerland. This article is an open access article distributed under the terms and conditions of the Creative Commons Attribution license (http://creativecommons.org/licenses/by/4.0/).
